# Therapeutic potential of combination medicinal mushrooms (NevG) in ischemic stroke: correlating motor function, cognitive recovery, and hippocampal integrity in MCAO rats

**DOI:** 10.3389/fphar.2025.1698883

**Published:** 2026-02-06

**Authors:** Nur Athirah Azlan, Misya Afiqah Noor Tuah, Nik Nasihah Nik Ramli, Hussin Muhammad, Zolkapli Eshak, Liao Peng, Chen Bo, Yatinesh Kumari, Imran Jazuli, Che Mohd Nasril Che Mohd Nasir, Syntyche Seow Ling Sing, Cheng Poh Guat, Hafizah Abdul Hamid, Zaw Myo Hein, Muhammad Danial Che Ramli

**Affiliations:** 1 School of Graduate Studies, Management and Science University, Shah Alam, Selangor, Malaysia; 2 Department of Diagnostic and Allied Health Science, Faculty of Health and Life Sciences, Management and Science University, Shah Alam, Selangor, Malaysia; 3 Toxicology and Pharmacology Unit, Herbal Medicine Research Centre, Institute for Medical Research, National Institutes of Health, Shah Alam, Selangor, Malaysia; 4 Department of Pharmacology and Chemistry, Faculty of Pharmacy, Puncak Alam, Selangor, Malaysia; 5 National Neuroscience Institute, Singapore, Singapore; 6 Neurological Disorder and Aging Research group (NDA), Neuroscience Research Strength (NRS), Jeffrey Cheah School of Medicine and Health Sciences, Monash University Malaysia, Bandar Sunway, Selangor, Malaysia; 7 Department of Anatomy and Physiology, Faculty of Medicine, Universiti Sultan Zainal Abidin, Kuala Terengganu, Terengganu, Malaysia; 8 Ganofarm R&D Sdn Bhd, Puchong, Selangor, Malaysia; 9 Department of Human Anatomy, Faculty of Health and Medical Sciences, University Putra Malaysia (UPM), Serdang, Selangor, Malaysia; 10 Department of Basic Medical Sciences, College of Medicine, Ajman University, Ajman, United Arab Emirates

**Keywords:** cognitive and sensorimotor function, ischemic stroke, medicinal mushrooms, middle cerebral artery occlusion, neuroprotection

## Abstract

**Background:**

Ischemic stroke is a major neurological disorder that is characterized by cognitive decline and sensorimotor impairment. Despite the potential of therapeutic effects and anti-inflammatory properties of medicinal mushrooms, most current research focuses on single-species effects rather than combined formulations. This gap highlights the need to investigate the potential of a combination of medicinal mushrooms named NevG, containing *Lignosus rhinocerus*, *Hericium erinaceus*, and *Ganoderma lucidum*, focusing on their therapeutic effects in the context of ischemic stroke.

**Methods:**

Forty adult male Sprague–Dawley rats were randomly assigned to five groups (*n = 8*): normal, MCAO-induced, and NevG in oral doses of 250 mg/kg, 500 mg/kg, and 1,000 mg/kg for 28 days. Ischemia was induced using the Koizumi method and confirmed through triphenyltetrazolium chloride (TTC) staining and mNSS scoring. Cognitive abilities were assessed using the Morris water maze and T-maze tests, and sensorimotor function was evaluated using the open-field, rotarod, and pole tests. Mechanistic analyses involved measuring anti-inflammatory serum cytokine pathways (TNF-α, IL-1β, IL-6, and NF-κβ).

**Results:**

NevG significantly reduced infarct volume by 32%–58% compared with the middle cerebral artery occlusion (MCAO) group (p < 0.05). Cognitive performance improved, with a 25%–46% reduction in the Morris water maze (MWM) escape latency (p < 0.01) and an increase in T-maze spontaneous alternation. Sensorimotor functions were enhanced, as evidenced by increases in the open-field test (OFT), rotarod retention time (p < 0.01), and decreased descent time in the pole test. The cresyl violet staining of the neurons in the hippocampus shows improvement in pyramidal cell counts, and the ultrastructural transmission electron microscope (TEM) analysis shows better preservation of the nucleus and mitochondria, intact myelin sheath, and improved axonal integrity. NevG significantly lowered the inflammatory serum cytokine level (p < 0.05) and promoted neuronal survival.

**Conclusion:**

NevG demonstrates significant neuroprotective effects in ischemic stroke, achieved through reduced neuroinflammation and improved neuronal survival, indicating its potential as a natural therapeutic agent.

## 1 Introduction

Stroke, also referred to as a cerebrovascular accident, involves brain damage due to an interrupted or inadequate blood supply to the brain tissue. Among the various types, ischemic stroke, resulting from a blockage in cerebral blood vessels, accounts for approximately 85% of all stroke cases globally. As of 2025, stroke continues to be the second leading cause of death and the third leading cause of disability worldwide, impacting over 15 million people each year, with more than 5.5 million fatalities. In Malaysia, stroke remains a major public health concern, ranking as the third leading cause of death and the primary cause of long-term disability (Tan and Venketasubramanian, 2022). This study focused on ischemic stroke as the most common type of stroke globally, compared to hemorrhagic and other variants (Chung, 2017). Recent national statistics suggest that over 50,000 new stroke cases occur annually, with 19,928 deaths linked to ischemic stroke (Feigin et al., 2024). The rising incidence underscores the urgent need for effective therapeutic approaches, particularly those focused on improving functional recovery after stroke. From a treatment standpoint, the development of neuroprotective agents has garnered significant attention from scientists and the pharmaceutical sector. Many preclinical studies have shown encouraging results with experimental compounds and interventions in rodent stroke models, highlighting the ongoing search for effective therapies (Nampoothiri et al., 2016).

NevG is a nutraceutical formulation comprising *Lignosus rhinocerotis* (*L. rhinocerotis*), *Hericium erinaceus* (*H. erinaceus*), and *Ganoderma lucidum* (*G. lucidum*) that is recognized for potent neuroprotective and anti-inflammatory activities. *L. rhinocerotis* is abundant in β-glucans, terpenoids, fungal immunomodulatory proteins, lectins, and ribosome-inactivating proteins, which contribute to neuroprotection and modulation of immune responses (Kamal et al., 2022). *H. erinaceus* synthesizes bioactive compounds such as erinacines and hericenones that stimulate nerve growth factor (NGF) production, promote neuronal repair, and reduce amyloid-β accumulation (Soumaya et al., 2025). In addition, its phenolic compounds and β-glucans exert strong anti-inflammatory effects (Garrab et al., 2019; Podkowa et al., 2020). *G. lucidum* contains ganoderic acids, polysaccharides, and glycoproteins, all of which demonstrate robust neuroprotective and immunomodulatory properties (Nandal et al., 2023).

Cognitive and motor impairments are among the most common and debilitating outcomes of ischemic stroke, significantly affecting survivors’ independence and quality of life. Cognitive impairment includes a wide range of deficits, such as memory loss, reduced attention, executive dysfunction, language disturbance, and visuospatial challenges (Elendu et al., 2023). These deficits often arise from stroke-induced damage to brain areas responsible for higher-order processing. Importantly, cognitive function is closely related to motor performance, as many aspects of motor control, such as planning, execution, and adaptation of movements, rely on intact cognitive systems. Damage to cortical and subcortical motor-related regions can lead to impaired motor coordination, affecting both gross and fine motor skills of the upper limbs. Furthermore, effective motor rehabilitation often depends on cognitive abilities, such as attention, working memory, and visuospatial processing, which are crucial for relearning lost motor skills and adapting to new motor tasks (D’Imperio et al., 2021). Therefore, understanding the interconnected nature of cognitive and motor dysfunction is essential for developing comprehensive rehabilitation strategies for stroke survivors.

Medicinal mushrooms contain diverse bioactive compounds, such as terpenoids, alkaloids, flavonoids, and polysaccharides, which are known for their therapeutic potential. Previous studies on *L. rhinocerotis*, *H. erinaceus*, and *G. lucidum* have demonstrated anti-neuroinflammatory effects, enhancement of NGF expression, reduction of infarction size, and improvements in cognitive and sensorimotor functions (Nallathamby et al., 2020; Pang et al., 2020; Panthakarn et al., 2025). However, little is known about the synergistic effects of mushroom combinations, particularly in ischemic stroke models. Evidence on their impact in middle cerebral artery occlusion (MCAO) rats, especially regarding infarction volume, behavioral outcomes, and inflammatory cytokine regulation, remains limited. Therefore, this study aims to evaluate the neuroprotective potential of NevG, a medicinal mushroom formulation, in improving post-stroke recovery through both functional and molecular outcomes.

## 2 Materials and methods

### 2.1 Animals

**FIGURE 1:**
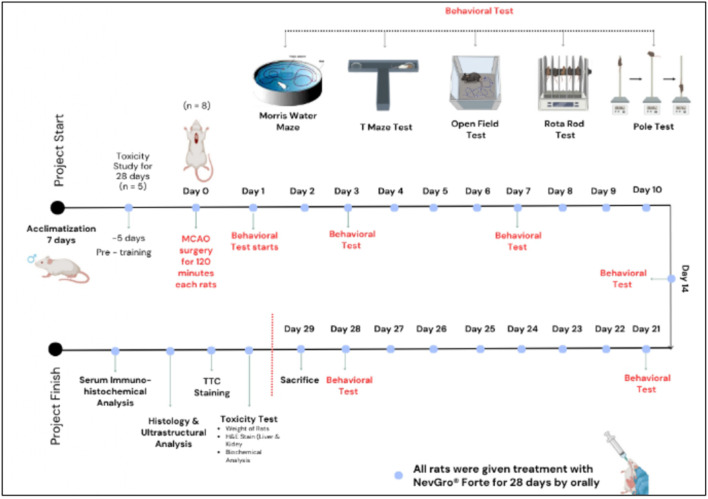
Diagram showing the timeline of behavioral testing for 28 days.

A total of 40 adult male Sprague–Dawley rats, aged 8 weeks and weighing 230–250 g, were obtained from the Institute for Medical Research (IMR), Ministry of Health, Malaysia. Animals were housed in groups of five per cage under standard laboratory conditions, including a 12-h light/dark cycle (lights on at 7:00 a.m.), controlled temperature (23 °C ± 2 °C), 55%–70%, and *ad libitum* access to food and water (Tang et al., 2021). All experimental procedures were conducted in accordance with protocols approved by the Animal Care and Use Committee of the Research Management Centre, Management and Science University. Efforts were made to minimize animal distress and to use the minimum number of animals necessary to achieve reliable results. The animals were allocated to the experimental groups as outlined in the treatment groups (*n = 8*) in Table 1. The study timeline from day 1 until day 28, from acclimatization to sacrifice, is illustrated in Figure 1.

**TABLE 1:** Treatment doses and groups.

Group	Animal (*n*)	Treatment	Experimental drug
1	8	Normal	Saline
2	8	MCAO induced	No treatment
3	8	MCAO + 250 mg/kg	250 mg/kg of NevG
4	8	MCAO + 500 mg/kg	500 mg/kg of NevG
5	8	MCAO + 1,000 mg/kg	1,000 mg/kg of NevG

### 2.2 Mushroom sample

The mushroom sample was authenticated by the Mushroom Research Centre, University of Malaya (UM), Malaysia. Identification was carried out based on morphological characteristics, and where applicable, supported by molecular analysis, following established methods reported in Malaysian mushroom-related studies. A voucher specimen has been deposited at the Mushroom Research Centre, UM, for future reference. The NevG product includes three different medicinal mushrooms that were obtained from GanoFarm R&D, Tanjung Sepat, Selangor, Malaysia.

Hericium erinaceus (Bull.; Fr.) Pers. is a medical mushroom that belongs to the division of Basidiomycota (Aphyllophoromycetideae, Hydnaceae). This mushroom is also known as Lion’s Mane, Monkey’s Head, Hedgehog Fungus, Satyr’s Beard, Pom Pom Blanc, Igelstachelbart, and Yamabushitake and is native to Europe, North America, and Asia (Lew et al., 2020). The second combination mushroom is known as the Tiger Milk mushroom (TMM), as, according to popular belief, the fruit bodies are often encountered in a forest, whereas the tiger has dripped its milk during feeding. The taxonomy of TMM is *Lignosus rhinoceros* (Cooke) Ryvarden, a polypore fungus belonging to the family Polyporaceae of the order Polyporales, Basidiomycota. This mushroom was also previously known as beteskismas, tish am ong, Pěti’ Aa, cendawan susu rimau (Malay), Ndurabi (Indonesia), how guikou or hurulingzhi (China), and hijiritake (Japan) (Vinjusha, 2021). The third medicinal mushroom is taxonomically known as *Ganoderma lucidum* (Curtis) P. Karst, and it is also called reishi and by the scientific binomial of *Lingzhi*. This mushroom belongs to the division of Basidiomycota, the order of Polyporales, and the family of Ganodermataceae. (Jin et al., 2025). In Indonesia and Australia, this species, known as *Ganoderma steyaertanum*, and other species in Asia are also known as *G. multipileum.*

### 2.3 Medicinal mushroom (NevG) extraction

The fresh fruiting bodies of the mushrooms (*H. erinaceus* and *G. lucidum*) and sclerotia (*L. rhinoceros*) were extracted freshly after being harvested from GanoFarm. The mushrooms were extracted using the hot water extraction method at a 10:1 ratio and spray-dried into powder. The individual mushroom extract powder was then mixed according to the NevG formula: NevG Lion’s Mane mushroom extract 55%, RespeRemè^®^ Tiger Milk mushroom extract 27%, and OriCidum^®^ Ganoderma lucidum extract 18%. The mixed NevG powder was then stored in a container at −20 °C until analysis.

### 2.4 Middle cerebral artery (MCAO)

**FIGURE 2:**
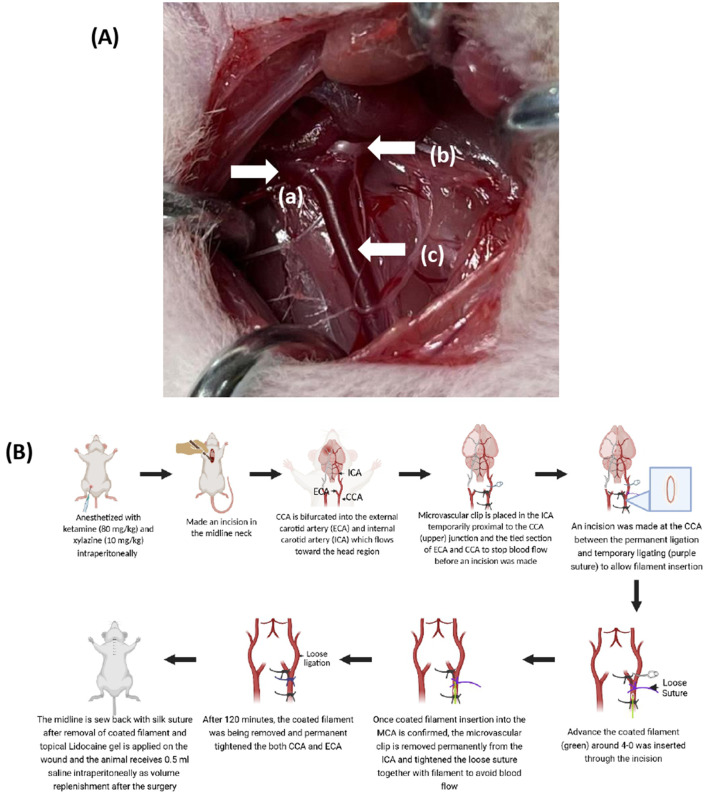
**(A)** Surgery of MCAO at the midline neck of rats, including the: (a) external carotid artery, (b) internal carotid artery, and (c) common carotid artery. **(B)** Steps of the Koizumi method from when the rats are anesthetized until the removal of the coated filament after 90 min.

The area beneath the chin was shaved and disinfected with an alcohol swab. A central incision was made between the jawbone and manubrium. The right common carotid artery (CCA) was carefully separated from the nearby vagus nerve to avoid nerve damage. The right external carotid artery (ECA) and internal carotid artery (ICA) were isolated from the surrounding fascia and adjacent nerves. The occipital and superior thyroid arteries (STA) were identified, separated, and cauterized. A silk suture (USP 4/0) was then tied around the ECA, positioned as far from the CCA bifurcation as possible. To protect the ICA, the surrounding structures were gently isolated and either cauterized or dissected using reverse-action scissors. At this point, the surgical technique diverges between the Koizumi and Longa methods of treatment. The Koizumi method was selected for this study, as shown in Figure 2 (Mulcahy et al., 2022). To limit cerebral blood flow (CBF), the CCA was permanently tied 5 mm away from the bifurcation, which can be monitored using laser Doppler flowmetry. A second loose collar suture was placed approximately 2 mm from the bifurcation. The ICA was temporarily blocked using a microvascular clamp. An arteriotomy was performed between the two sutures using reverse-action scissors. A silicone-coated filament was inserted through the arteriotomy and advanced through the right ICA until it reached the microvascular clamp of the aneurysm. The clamp was then removed, allowing the filament to be gently advanced toward the MCA. After 90 min of occlusion, the intraluminal filament was partially withdrawn to initiate reperfusion, thus establishing a transient MCAO model. During occlusion, the loose collar suture was tightened to secure the filament in place. After a total occlusion time of 120 min, the filament was retracted to the bifurcation level, and the microvascular clamp was reapplied to the ICA. The filament was then fully withdrawn to prevent backflow through the arteriotomy site, and the collar suture was tightened around the CCA. Finally, the microvascular clamp was removed from the anastomosis. The entire surgical procedure lasted approximately 20 min (Mulcahy et al., 2022).

### 2.5 Triphenyltetrazolium chloride (TTC)

TTC staining is one of the confirmation studies for ischemic stroke. Twenty-four hours after MCAO, the brain was collected to assess the infarct volume. The brain tissue was frozen at −20 °C for 30 min and then sliced into six evenly spaced coronal sections, each approximately 2 mm thick. The sections were stained in 2% TTC at 37 °C for 30 min and then soaked in 4% paraformaldehyde for 24 h. Images of the stained brain were taken and analyzed using ImageJ software to measure the infarct area. In TTC-stained sections, infarcted (damaged) areas appear white, while healthy tissue appears red. A ruler was used for accurate sizing, and the infarct percentage was calculated using the formula: (infarct area/total brain area) × 100% (Liepinsh et al., 2022).

### 2.6 Neurological assessment

A series of behavioral tests was conducted to assess neurological impairments following ischemic stroke in rats. Before undergoing MCAO surgery, all animals participated in a 1-week training session to establish baseline performance levels. Behavioral evaluations began 24 h post-MCAO and continued over a 28-day period. Each test was recorded on video using a mobile phone at designated times. The assessments, which included the T-maze, Morris water maze, pole test, open-field test, and rotarod, were performed on days 1, 3, 7, 14, 21, and 28 after MCAO, respectively.

#### 2.6.1 Evaluation of modified neurological severity scores (mNSS)

All rats underwent a behavioral test called the modified neurological severity score (mNSS) 2 h after MCAO to confirm the presence of ischemic stroke. The scoring was assessed on days 1, 3, 7, 14, 21, and 28. This scoring system includes 14 items that assess motor skills, sensory function (such as vision and touch), balance, and reflexes. Each abnormal response scores one point. The total score reflects the severity of the stroke: 0, no impairment; 1–4, mild; 5–9, moderate; and 10–14, severe. A higher score indicates more serious neurological damage (Geng et al., 2021).

#### 2.6.2 Cognitive testing

##### 2.6.2.1 Morris water maze (MWM)

Cognitive function was assessed using the Morris water maze and T-maze tests. In the Morris water maze, a large circular pool was divided into four quadrants (NE, NW, SE, and SW), with a hidden platform placed 2 cm below the water surface. Rats had 90 s to find the platform; if they failed within 180 s, they were guided to it and allowed to stay for 60 s to learn its location. After each trial, they remained on the platform for 30 s to reinforce memory. Testing occurred from day 7 to day 28 after stroke, tracking swim speed and time to reach the platform (Sayed et al., 2020).

##### 2.6.2.2 T-maze test

The T-maze test was used to assess working and spatial memory in rats. The T-shaped maze included a start area and two arms. Before and after inducing the stroke, rats underwent trials to evaluate memory performance. Each trial had two phases: a “sample” phase in which one arm was blocked, and the rat was guided to the open arm with a food reward, and a “run” phase in which both arms were open. Choosing the correct (green) arm resulted in a reward, while the wrong (red) arm led to a mild punishment. Trials were recorded for 2 min, tracking correct choices and speed of learning. The actual time recorded was 2 min per test (d’Isa et al., 2021).

#### 2.6.3 Sensorimotor skill test

##### 2.6.3.1 Open-field test (OFT)

The OFT was used to assess emotional behavior in rats using a 50 cm × 50 cm × 38 cm box with a computerized tracking system. The floor was divided into 9 inner (center) zones and 16 outer zones. Normal rats tend to explore the inner zone, while stroke rats stay near the outer edges. Tests were done on days 1, 3, 7, 14, 21, and 28 after surgery. Rats were familiarized with the chamber 2 h before testing and observed for 10 min. Time spent in each zone and total distance traveled were analyzed using ANY-maze software (Vuralli et al., 2019).

##### 2.6.3.2 Rotarod test

The rotarod test was used to assess balance and motor coordination in MCAO rats. The rotating rod spun up to 20 rpm, with a soft platform 18 cm below to prevent injury. Rats were trained for 5 days (three 30-s trials daily) before MCAO, continuing until they could stay on the rod for at least 1 min. Testing was done from day 7 to 28 after stroke in three trials per day. During tests, the rod gradually accelerated from 4 rpm to 20 rpm over 2 min, and the time before the rats fell was recorded (Srivastava et al., 2020).

##### 2.6.3.3 Pole test

The pole test is used to assess locomotor function in rodents. During the test, the rat was placed on a wooden pole with its head up. The time taken to descend (tD) and the time to perform a turn (tT) were recorded. Time for tD indicates the total time from the beginning until the rat fell down, and tT refers to the time when the rat turned its head down. If a rat turned but fell halfway, the total time until it reached the floor was noted. Animals underwent 2 days of training, with five trials per day. The final score is the average of the five trials from each session (Mustafa et al., 2020).

### 2.7 Histological analysis

#### 2.7.1 Cresyl violet staining

Ischemic rat brain sections were fixed in formalin and embedded in paraffin. They were then deparaffinized with xylene and rehydrated using a graded series of alcohol. The sections were stained with 0.1% cresyl violet for 3–5 min, rinsed in distilled water, and differentiated in 95% alcohol. Subsequently, the tissues were dehydrated in absolute serial ethanol, cleared in xylene, and mounted with DPX mounting medium onto gelatinized microscopy slides under cover glass (Lee et al., 2021).

#### 2.7.2 Transmission electron microscope (TEM)

A small amount (approximately 1 mm^3^) of brain tissue from the hippocampus area, CA1, CA2, CA3, and the dentate gyrus (DG) was fixed in 2.5% glutaraldehyde at 4 °C for 2–4 h. The samples were rinsed three times with 0.1 M phosphate buffer for 15 min each and then post-fixed in 1% osmium tetroxide at 4 °C for 2 h. Dehydration was carried out sequentially using 30%. 50%, 70%, 80%, 95%, and 100% ethanol, with each step lasting 15 min. This was followed by three washes in 100% propylene oxide and embedding at resin at a 1:1 ratio for 2 h at 37 °C, followed by a 1:2 ratio for 5 h at 37 °C and, finally, with pure embedding resin overnight at 37 °C. The embedded samples were polymerized at 60 °C for 48 h. Once polymerized, the resin blocks were trimmed and sectioned using an ultramicrotome to obtain semi-thin and ultra-thin sections. The sections were stained with 2% uranyl acetate in saturated alcohol for 8 min, rinsed three times in 70% ethanol, followed by three rinses in ultrapure water, and gently blotted with filter paper. Finally, the sections were examined and photographed using a transmission electron microscope (Yang et al., 2022).

### 2.8 Immuno-biochemical analysis

Blood was collected from the rat tail and centrifuged at 3,500 rpm/min for 10 min at room temperature, and the serum was extracted. A rat ELISA kit was used to measure the protein level of TNF-α, IL-1β, IL-6, and NF-κβ according to the manufacturer’s instructions from Bioassay Technology Laboratory (Bioassay Technology Laboratory). The graph of standard was conducted, and the results were analyzed using GraphPad Prism 10.2.3 software. The results are expressed as ng/mL protein (Zhong et al., 2024).

### 2.9 Data analysis

All statistical analyses were performed using GraphPad Prism 10.2.3 software. The data collected from these assessments were analyzed using one-way ANOVA with Tukey’s post-hoc test applied for multiple comparisons. Values are expressed as mean ± standard error of the mean (SEM), with statistical significance set at p ≤ 0.05.

## 3 Results

### 3.1 NevG shows a reduction in the brain infarction area using 2,3,5-triphenyltetrazolium chloride (TTC) staining

**FIGURE 3:**
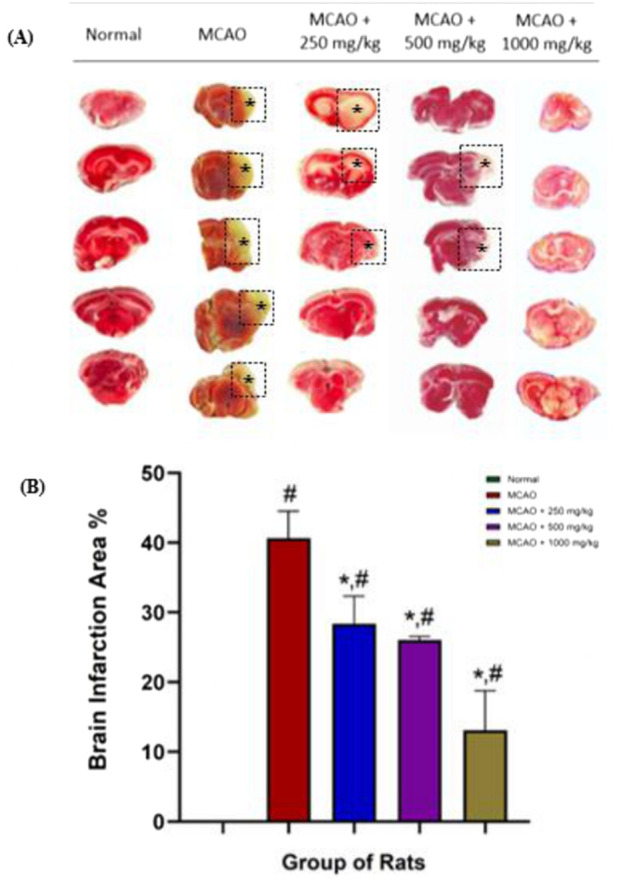
Coronal sections of representative ischemic brain sections of the cerebral area at 2 mm after the level of bregma of Sprague–Dawley (SD) rats administered with NevG and the infarct area of TTC staining with the bar graph of brain infarction area %. TTC serially sectioned coronal brain tissues indicate that the white area is the infarction and the red area is the healthy brain, as shown in **(A)**. Bar chart of the brain infarction area %. Significantly reduced infarct volume when compared with the MCAO group, shown in **(B)**. The data are presented as mean ± SEM, p < 0.05. * indicates p < 0.05 toward the MCAO group; # indicates p < 0.05 toward the normal group when applied 28 days after reperfusion.

TTC staining revealed significant cerebral infarction in the untreated MCAO group, with approximately 40% of the tissue affected, indicating severe ischemic damage (Santo et al., 2023). Based on the picture shown in Figure 3A, infarcted regions appeared white, whereas viable brain tissue was stained red. Treatment with NevG resulted in a clear dose-dependent reduction in infarct size, as the 250 mg/kg group experienced a moderate decrease to 28%, and the 500 mg/kg group showed further improvement with a 26% infarction area. The 1,000 mg/kg group demonstrated the most substantial protection, reducing infarction to 13%, as reported by the bar graph in Figure 3B. The increased presence of red-stained viable areas in the higher-dose groups suggests increased mitochondrial activity and preserved tissue integrity. No toxicity was detected in any treatment group, indicating that NevG offers effective, dose-dependent therapeutic effects following MCAO. The MCAO group exhibited extensive infarction, indicative of severe ischemic damage at 24 h post-occlusion. In contrast, NevG-treated groups demonstrated a dose-dependent reduction in infarct volume, with up to a 29% decrease relative to the MCAO group. The 1,000 mg/kg dose produced the most pronounced neuroprotective effect against post-ischemic injury.

### 3.2 Behavioral analysis

#### 3.2.1 Modified neurological severity score (mNSS)

**FIGURE 4:**
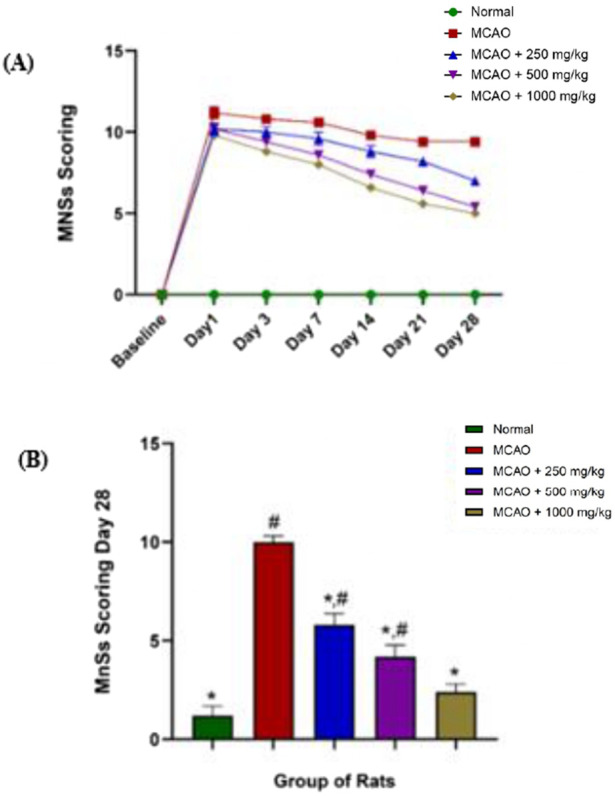
Neural function following MCAO was assessed using the modified mNSS. The severity scores were calculated from 0 to 14, and the trend over days 1, 3, 7, 14, 21, and 28 is shown in **(A)**. The MCAO model group scored significantly higher than the control group and the NevG groups, as shown in **(B)**. The data are presented as mean ± SEM, p < 0.05. * indicates p < 0.05 toward the MCAO group; # indicates p < 0.05 toward the normal group.

Neurological scores progressively declined from day 1 post-surgery to day 28 following treatment. Statistical analysis of the 14-point mNSS revealed significant differences among groups over time, as shown in Figure 4A. The MCAO group exhibited severe neurological deficits (10.60 ± 1.788) compared with both the normal (1.200 ± 0.489) and the NevG-treated groups. Administration of 250 mg/kg of NevG resulted in moderate improvement (7.543 ± 1.313), whereas the 500 mg/kg (5.971 ± 1.210) and 1,000 mg/kg (5.514 ± 1.196) groups significantly reduced mNSS scores relative to the MCAO group on days 1, 3, 7, 14, 21, and 28, indicating a clear therapeutic benefit for motor function. Recovery was dose-dependent, with the 1,000 mg/kg group achieving the most substantial improvement by day 28 Figure 4B.

#### 3.2.2 Cognitive testing

##### 3.2.2.1 Morris water maze

**FIGURE 5:**
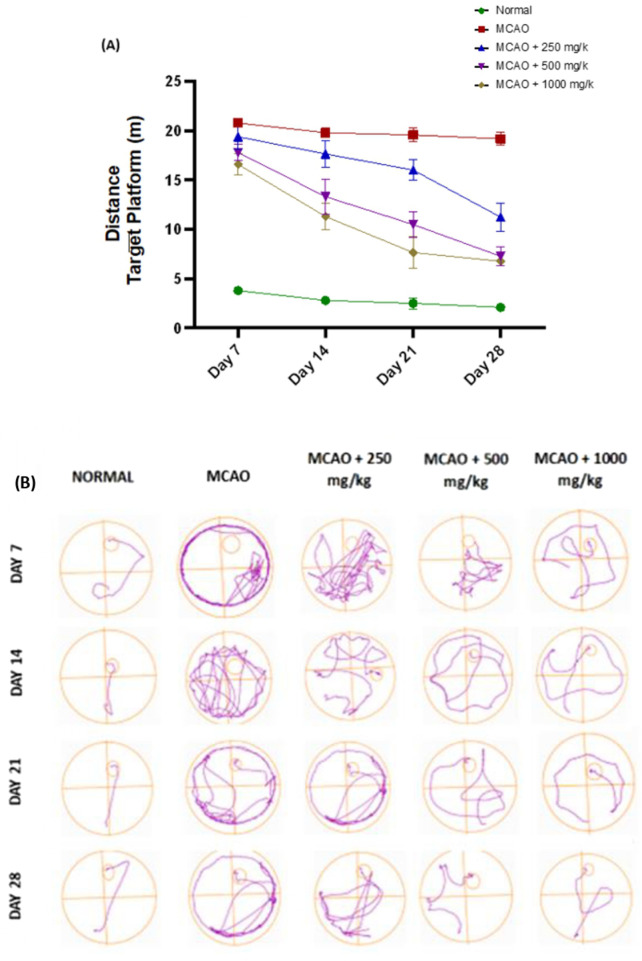
MCAO caused a cognitive decline in stroke rats. Line graph **(A)** shows the distance rats traveled to the target platform (meter) during the navigation test on days 7, 14, 21, and 28 post-operation, and **(B)** shows the swim traces of the rats following the groups of treatments.

In the Morris water maze (MWM) test, repeated measured of ANOVA revealed significant cognitive impairments in the MCAO group, with erratic prolonged escape latencies (8.073 ± 0.386, p < 0.0001) in Figure 5A and swim path pattern Figure 5B revealed that MCAO rats took longer path length and longer time to locate the submerged hidden platform reflecting severe impairment of spatial working memory and executive function due to ischemic injury. In contrast, the normal group demonstrated efficient navigation (74.17 ± 2.316), indicating intact spatial memory. Treatment with NevG showed dose-dependent improvement in enhanced spatial learning starting from day 14 post-ischemia. The lower dose, 250 mg/kg group (36.37 ± 7.313), exhibited some recovery, while the medium-dose group, 500 mg/kg group (41.62 ± 6.896), showed more consistent results on day 21. The group receiving the highest dose, 1,000 mg/kg (52.01 ± 10.43), of NevG needed less time to reach the target platform on day 21, suggesting improved memory and indicating sustained recovery and enhanced long-term neuroadaptation.

##### 3.2.2.2 T-maze test

**FIGURE 6:**
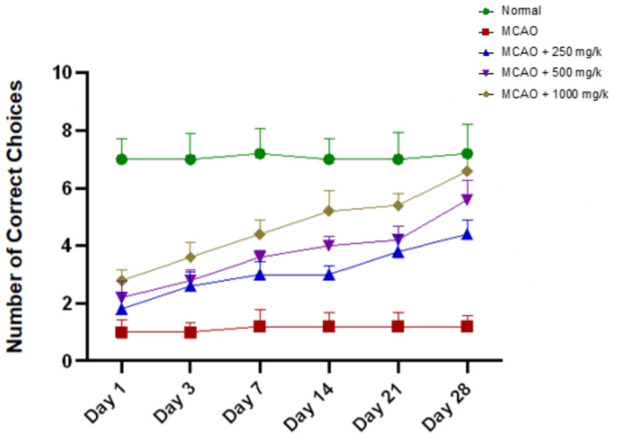
The effect of ischemic stroke following MCAO on the number of left and right choices in the T-maze was assessed on days 1, 3, 7, 14, 21, and 28. The correct left and right choices were recorded and are presented as a line graph. Results are expressed as the mean ± SEM, p < 0.05.

Cognitive assessment using the T-maze to assess performance of the correct choice of arm (right turn) revealed significant impairment in the MCAO group, which shows the MCAO group has fewer correct choices (1.33 ± 0.02) than the normal group (7.15 ± 0.04). NevG treatment improved alternation rates in a dose-dependent manner; however, the low-dose 250 mg/kg (4.65 ± 0.59) and the medium-dose 500 mg/kg (3.95 ± 0.43) groups showed that there was no significant difference on day 3, indicating only minor early improvement, and showing a significant difference from the start from day 7. The highest dose group, 1,000 mg/kg (3.40 ± 0.17), showed significantly more alteration to the correct choice than the MCAO group. Overall, these findings confirm that NevGro® Forte treatment improved post-stroke cognitive function in MCAO rats, with the greatest efficacy observed at 1,000 mg/kg, followed by 500 mg/kg, while lower doses offered partial benefit, as shown in Figure 6.

#### 3.2.3 Sensorimotor testing

##### 3.2.3.1 Open-field test (OFT)

**FIGURE 7:**
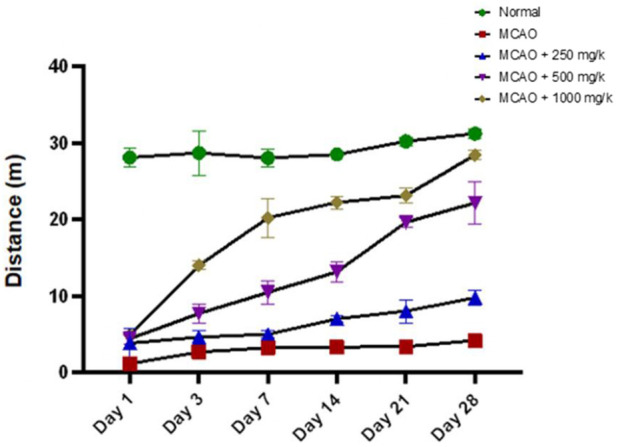
The performance of rats in the open-field test was evaluated by measuring the total distance traveled over a 10-min period. Data are presented as mean ± SEM, p < 0.05.

There are significant deficits in the MCAO group (2.233 ± 0.254), compared to the normal group (p < 0.05), as shown in Figure 7. Normal rats maintained consistent locomotor activity, while MCAO rats remained largely immobile across all time points. NevG treatment showed dose-dependent improvements. The 250 mg/kg group (5.616 ± 0.973) showed minor and non-significant locomotor gains. In contrast, the 500 mg/kg and 1,000 mg/kg groups significantly increased mobility (12.53 ± 2.841) and (17.68 ± 3.272), respectively, with the 1,000 mg/kg group approaching normal levels by day 28. Early-phase (days 1–3) recovery was limited, but by day 3, the 500 mg/kg and 1,000 mg/kg groups showed significant mobility gains, whereas the 250 mg/kg group lagged. By days 21–28, locomotor recovery plateaued, with the 1,000 mg/kg group achieving activity levels statistically comparable to the normal group, while the 500 mg/kg group also showed notable improvement. The 250 mg/kg group, however, maintained limited recovery, reflecting the suboptimal efficacy at lower doses.

##### 3.2.3.2 Rotarod test

**FIGURE 8:**
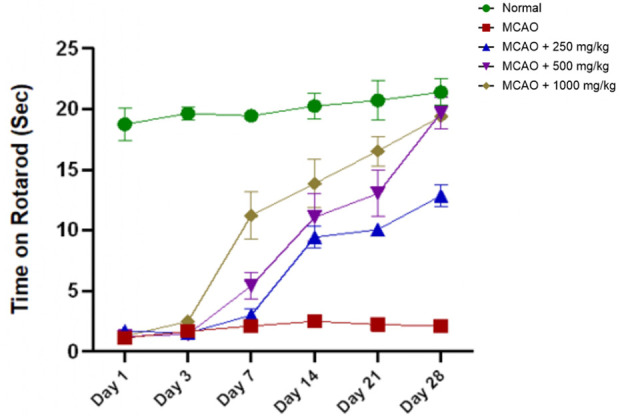
Rotarod tests were evaluated on days 1, 7, 14, 21, and 28 following stroke to assess motor coordination and balance in the subjects. Data are presented as mean ± SEM, p < 0.05.

The MCAO group showed significantly reduced performance in terms of maintaining their balance on the rotarod (8.07 ± 0.39). No significant deficits were observed until day 3 compared to the normal group (74.17 ± 2.14) in seconds, as shown in Figure 8, reflecting severe motor impairment. NevG treatment led to dose-dependent improvements of the low-dose 250 mg/kg (36.37 ± 7.31), medium-dose 500 mg/kg (41.61 ± 6.90), and high-dose 1,000 mg/kg (52.01 ± 10.43) groups observed on day 7. Although none of the treatment groups fully recovered to normal levels, higher doses significantly improved motor function. Overall, with increasing time, the rotarod findings support a dose-dependent effect of NevG in restoring motor coordination post-stroke, with the 500 mg/kg and 1,000 mg/kg doses offering the most robust recovery outcomes reached and stable by days 21.

##### 3.2.3.3 Pole test

**FIGURE 9:**
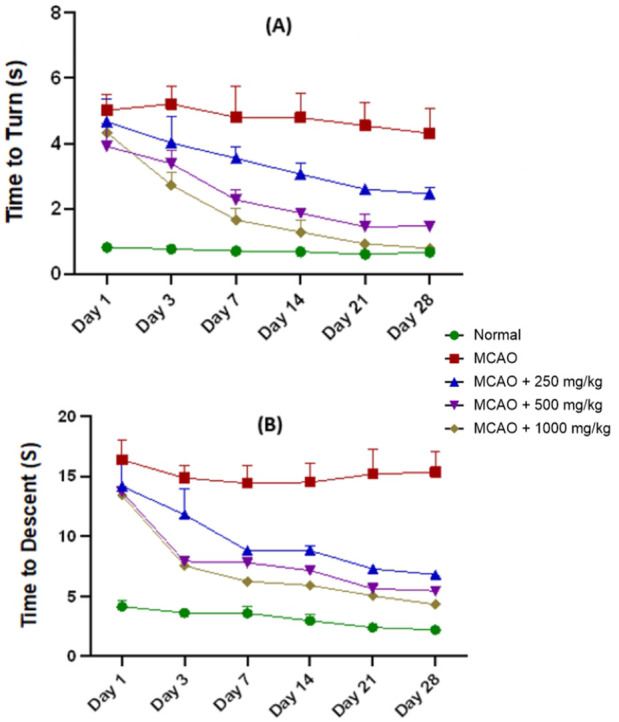
Assessment of coordinative abilities of the pole test. **(A)** Latency of the rat to turn down and **(B)** time of the rat to fall were recorded in seconds on days 1, 3, 7, 14, 21, and 28. Statistical significance testing was analyzed by one-way ANOVA, and data are presented as mean ± SEM, p < 0.05.

The pole test revealed sensorimotor impairments persisting for up to 28 days after ischemic injury. In terms of time to turn (tT), the MCAO group (4.82 ± 0.22) experienced a significantly longer delay in time to move and turn the body than the normal group (0.71 ± 0.03). NevG improved tT in a dose-dependent manner, as the 250 mg/kg group (2.98 ± 0.67) had only mild improvement and showed noticeable delays, while the 500 mg/kg group (2.32 ± 0.52) had a moderate improvement with significant recovery beginning on days 7–14. The 1,000 mg/kg group (1.76 ± 0.62) showed strong recovery over time and approached almost normal values by days 21–28, as shown in Figure 9A. Regarding time to descend (tD), the MCAO group (15.04 ± 0.24) showed a significantly prolonged descent over time, which reflects a major change of motor impairment, balance, and bradykinesia compared to normal rats (1.91 ± 0.06). NevG significantly reduced tD in a dose-dependent manner, as the 250 mg/kg group (9.61 ± 1.16, P = 0.0046) showed only partial recovery, indicating limited sensorimotor restoration. The 500 mg/kg group (7.95 ± 1.23) had better coordination and enhanced motor control, and the 1,000 mg/kg group (7.09 ± 1.34) showed the most effect, with a substantial restoration of balance and coordination, as shown in Figure 9B. Over the 28-day period, MCAO rats exhibited severe deficits on days 1–3, with notable recovery in the 500 mg/kg and 1,000 mg/kg groups beginning on days 7–14. By days 21–28, the 1,000 mg/kg group nearly reached normal performance, whereas the 250 mg/kg group showed moderate but slower progress.

### 3.3 Histological analysis

#### 3.3.1 Medicinal mushrooms (NevG) improve neuronal survival in MCAO rats

**FIGURE 10:**
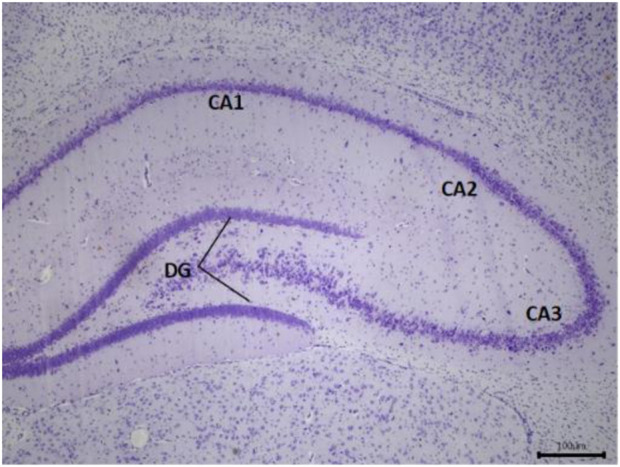
Representative photomicrograph of the hippocampal formation in a normal rat, illustrating the CA1, CA2, CA3, and DG regions at ×4 magnification.

**FIGURE 11:**
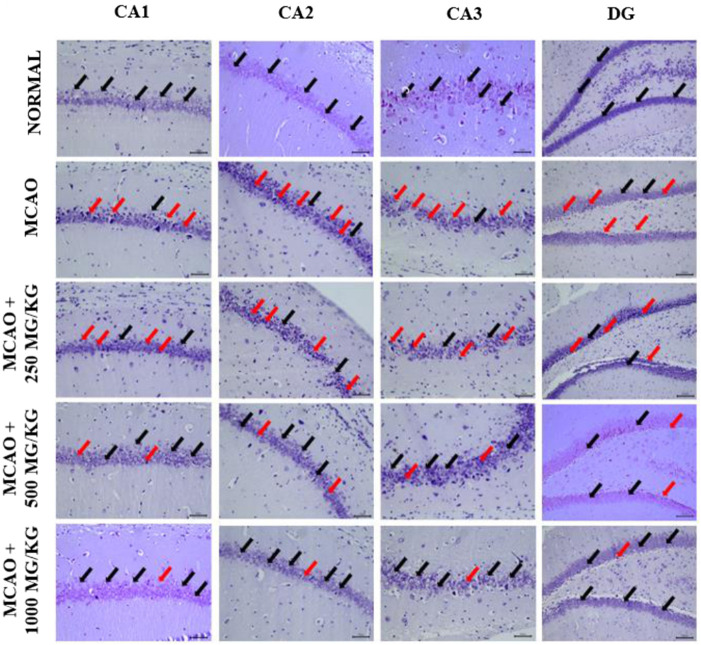
Representative photomicrographs of the pyramidal cells in the CA1, CA2, CA3, and DG subregions of the hippocampus in the brains of different groups of rats. The normal group shows many viable and healthy pyramidal cells densely. The MCAO group shows a very thin layer of degenerated pyramidal cells. The low-dose group has slightly dense layers of viable pyramidal cells and some degenerated cells. The medium-dose group has many viable pyramidal cells arranged in a thick layer and few degenerated cells. The highest dose group has improved viable, healthy pyramidal cells in a compact arrangement similar to the normal group. The black arrow shows normal, viable, and healthy pyramidal neurons, while the red arrows point at degenerated pyramidal neurons.

**FIGURE 12:**
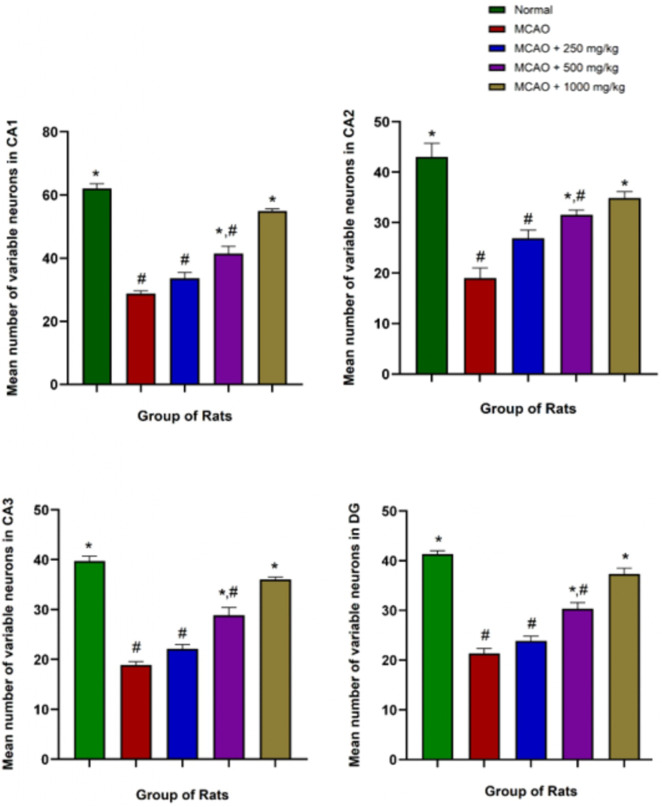
The number of variable neurons of the stratum pyramidale counted in each region, where the trend of the graph shows the number of viable neurons increasing throughout the higher dosage of the treatments given. * indicates p < 0.05 toward the MCAO group; # indicates p < 0.05 toward the normal group. Statistical significance testing was analyzed by one-way ANOVA, and data are presented as mean ± SEM, p < 0.05.

The protective effects of NevG on ischemic brain injury were further evaluated through cresyl violet (CV) for neuronal count, and the mean of variable neurons and whole hippocampus are presented in Figure 10. Compared to the normal group (CA1-DG), which showed healthy, intact cells, denser with higher neuronal counts, the MCAO-only group showed many pyknotic cells in the pyramidal cell layer, fewer neurons, and the least dense of all (CA1-DG) subfields of the hippocampus. In the groups treated with 250 mg/kg, 500 mg/kg, and 1,000 mg/kg of NevG for 28 days starting from day 1 after MCAO, there were surviving pyramidal neurons in all subfields of the hippocampus, although cell death was observed in all subfields at in the low- and medium-dose groups (250 mg/kg and 500 mg/kg). The damage in the group receiving the highest dose of NevG suggested therapeutic effects against the neuronal injury because the neuronal cell degeneration was almost similar to that of the normal group. The representative photomicrographs of the pyramidal cells in the CA1, CA2, CA3, and DG subregions of the hippocampus in the brain of the different groups of rats are presented in Figure 11, and the means of pyramidal viable neuron counts are presented in Figure 12.

One-way ANOVA showed that the pyramidal cells in the CA1 subregion of the normal group (62.11 ± 1.457) exhibited healthy neuronal morphology. In contrast, the MCAO group (28.78 ± 0.889) showed a significant reduction in viable neurons, with evident signs of pyknotic nuclei. Treatment with NevG resulted in a dose-dependent increase in CA1 neuronal counts, with doses of 250 mg/kg (33.67 ± 1.836), 500 mg/kg (41.44 ± 2.328), and 1,000 mg/kg (54.89 ± 0.676) progressively restoring pyramidal cell numbers closer to the normal levels. In the CA2 subregion, normal rats (43.00 ± 2.715) had significantly higher counts of healthy neurons than the MCAO group (19.00 ± 1.681). The 250 mg/kg group (26.89 ± 1.681) did not show a significant improvement from MCAO, whereas the 500 mg/kg (31.56 ± 0.949) and 1,000 mg/kg (34.89 ± 1.252) groups demonstrated notable increases in viable pyramidal neurons. Similarly, the CA3 subregion exhibited considerable neuronal loss in the MCAO group (18.89 ± 0.676) compared to that in normal rats (39.78 ± 0.990). NevG treatment improved neuronal survival in a dose-dependent manner, with 250 mg/kg (22.11 ± 0.868), 500 mg/kg (28.89 ± 1.544), and 1,000 mg/kg (36.00 ± 0.509) showing progressive restoration of intact pyramidal cells. In the DG region, the normal group had (41.33 ± 0.693) viable neurons per field, whereas the MCAO group had significantly fewer viable neurons (21.33 ± 1.018). NevG treatment increased DG neuronal density across all doses: 250 mg/kg (23.89 ± 0.909), 500 mg/kg (30.33 ± 1.262), and 1,000 mg/kg (37.33 ± 1.171), indicating consistent improvement of pyramidal cell counts. Across all hippocampal subregions, the progressive increase in neuronal count with the highest NevG doses demonstrates a clear dose-dependent therapeutic effect in ischemic injury.

#### 3.3.2 Effects of NevG on the ultrastructure changes in the nucleus, mitochondria, myelin, and axons of the hippocampus

**FIGURE 13:**
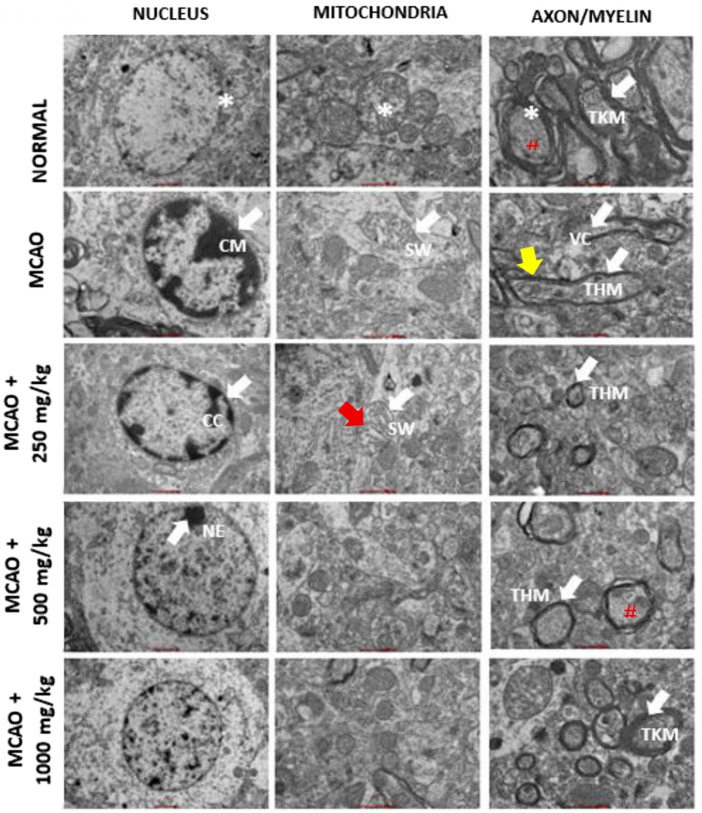
Representative TEM images of the nucleus, mitochondria, and axons from a rat brain hippocampus. The images of the hippocampus were observed 28 days after post-ischemic stroke, except for the MCAO group, which was taken after 24 h. TKM, thick myelin; THM, thin myelin; CM, chromatin margination; CC, condensed chromatin; NE, nuclear envelope; SW, swelling; VC, vacuolization. The normal cell is marked by *. The pound sign (#) represents a myelinated axon on the outside of the myelin sheath. The red arrow indicates a mitochondrion with membrane rupture and cristae fragmentation. The yellow arrow indicates an axon separated from the myelin sheath. In the micrographs, the scale bar corresponds to approximately 1 μm for the nucleus and 500 nm for the mitochondria, myelin, and axon images.

**FIGURE 14:**
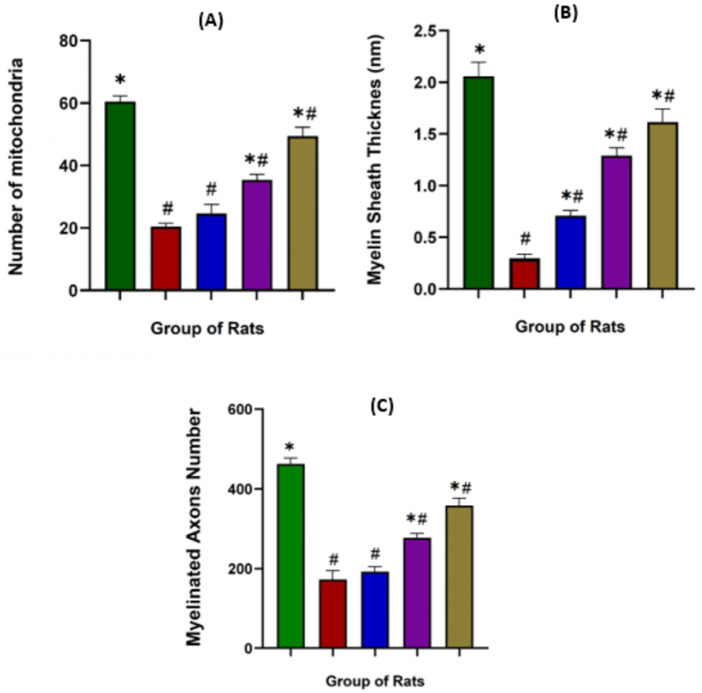
Effects of NevGro® Forte on the ultrastructure of the numbers of mitochondria, myelin sheath thickness, and myelinated axons in the corpus callosum (hippocampus) of MCAO rats evaluated by TEM. **(A)** The bar chart shows the number of mitochondria in each rat group. **(B)** Quantification of the myelin sheath thickness (nm) and **(C)** the number of myelinated total axons. Data are expressed as mean ± SEM. * indicates p < 0.05 toward the MCAO group; # indicates p < 0.05 toward the normal group.

In the normal hippocampus, neuronal nuclei appeared large and oval with a distinct nuclear membrane. The mitochondria displayed an oval shape enclosed by a double membrane with well-defined cristae, myelin sheaths consisted of compact, concentric layers with substantial thickness, and axons exhibited longitudinally arranged microtubules and neurofilaments. In contrast, marked pathological alterations were observed in the MCAO group, including shrunken nuclei, irregular nuclear membranes, and chromatin margination (CM). Mitochondria were swollen (SW), and perivascular edema with vacuolation was evident. Myelin sheaths appeared thinned and fragmented, while axons demonstrated swelling and tissue vacuolization (VC), and partial detachment from the myelin sheath, as shown in Figure 13. NevG treatment attenuated these ultrastructural abnormalities in a dose-dependent manner. The organelles from the 1,000 mg/kg group show the greatest preservation of cellular architecture, including nuclear morphology, mitochondrial structure, myelin sheath thickness, and axonal integrity. The quantitative analysis is shown in Figure 14. White arrows indicate the chromatin margination, condensed chromatin, and nuclear envelope observed in the nucleus. In mitochondria, the white arrow shows a swelling, and the red arrow shows mitochondria with membrane rupture and cristae fragmentation. The white arrow in the axon and myelin indicates a thick and thin layer of myelin and also its vacuolization. The (#) indicates the axon, and the yellow arrow indicates an axon separated from the myelin sheath. Following treatment, the ultrastructural characteristics of the hippocampal tissue showed marked improvements across the treatment groups, indicating a positive restorative effect at the cellular and subcellular levels.

Transmission electron microscopy (TEM) analysis revealed significant ultrastructural damage in the MCAO group compared to normal rats, which affects the mitochondrial count, myelin sheath thickness, and number of myelinated axons. Based on the data analysis of one-way ANOVA, the number of mitochondria was markedly lower in MCAO rats (20.40 ± 1.122) than in the normal group (60.40 ± 1.860), indicating severe energy dysfunction following ischemia. Treatment with NevG restored mitochondrial numbers from the 250 mg/kg (24.60 ± 2.909) to the 500 mg/kg (35.40 ± 1.749) to the 1,000 mg/kg (49.40 ± 2.857) groups, suggesting enhanced cellular metabolic stability after treatments. The thickness of myelin sheath was significantly reduced in the MCAO group (0.297 ± 0.041) compared to the normal group (2.058 ± 0.136), indicating demyelination and axonal damage. Treatment with NevG progressively increased the myelin thickness in the 250 mg/kg (0.708 ± 0.052), 500 mg/kg (1.290 ± 0.077), and 1,000 mg/kg (1.614 ± 0.129) groups, suggesting partial structural recovery and reduced white matter degeneration. Similarly, the number of myelinated axons significantly decreased in the MCAO group (172.8 ± 22.29) compared to the normal group (463 ± 14.26). NevG treatment increased axonal preservation in a dose-dependent manner, with the 250 mg/kg (191.8 ± 13.22), 500 mg/kg (276.8 ± 11.59), and 1,000 mg/kg (358 ± 17.40) groups showing progressive restoration. Altogether, these ultrastructural findings confirm that NevG mitigates mitochondrial loss, preserves myelin integrity, and maintains axonal structure after ischemic injury, with the strongest protective effects observed at 1,000 mg/kg.

### 3.4 Effects of NevG on the levels of TNF-α, IL-1β, IL-6, and NF-κβ in the blood serum of ischemic rats after MCAO

**FIGURE 15:**
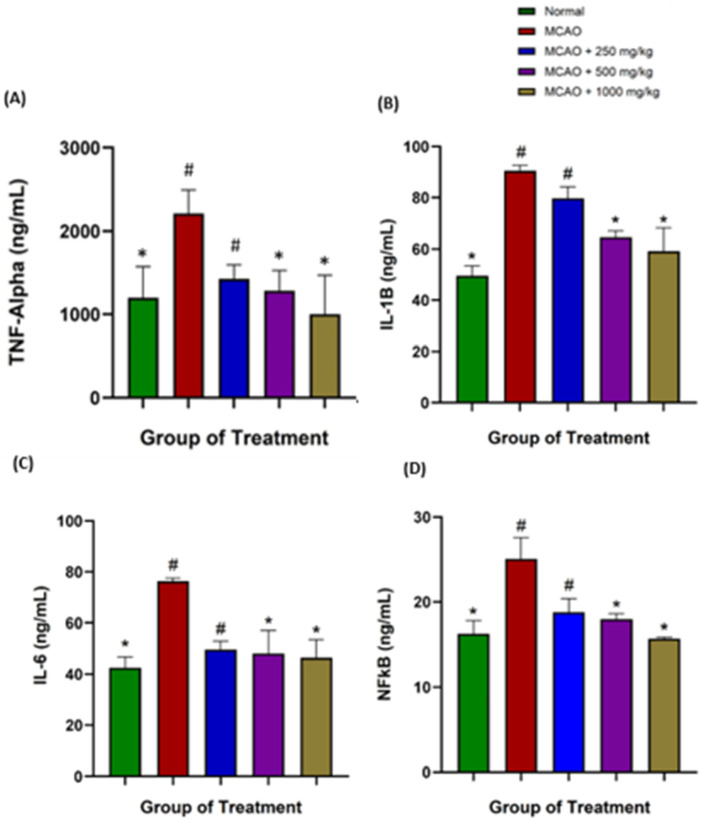
Bar graph represents the inflammatory cytokines using the ELISA test, as follows: The anti-inflammatory cytokines include (*n = 8*) **(A)** TNF-α, **(B)** IL-1β, **(C)** IL-6, and **(D)** NF-κβ. The data are represented as mean ± SEM, p < 0.05. * indicates p < 0.05 toward the MCAO group; # indicates p < 0.05 toward the normal group.

Assessment of serum pro-inflammatory cytokine pathways was conducted after 28 days of NevG treatment to determine its potential anti-inflammatory effects using ELISA (Bioassay Technology Laboratory). The levels of TNF-α, IL-1β, IL-6, and NF-κβ demonstrated a significant reduction in all NevG-treated groups compared with the MCAO group (p < 0.05) as shown in Figure 15. NevG administration effectively attenuated ischemia-induced cytokine upregulation, indicating suppression of MCAO-associated protein increase and supporting its anti-inflammatory properties. After MCAO, the levels of all measured pro-inflammatory cytokines markedly increased, indicating a strong systemic inflammatory response. TNF-α levels increased significantly from baseline (1,333 ± 56.69 pg/mL) to much higher levels in the MCAO group (2,324 ± 64.46 pg/mL). Treatment with NevG reduced TNF-α levels in a dose-dependent manner, with values of 250 mg/kg (1,537 ± 39.85 pg/mL), 500 mg/kg (1,394 ± 30.32 pg/mL), and 1,000 mg/kg (1,302 ± 48.58 pg/mL), indicating a gradual decrease in early inflammatory signaling. Similarly, IL-1β levels were elevated in MCAO rats (90.50 ± 2.22 pg/mL) compared to those in normal controls (49.50 ± 3.98 pg/mL). All rats treated with NevG had significantly decreased levels of IL-1β: 250 mg/kg (79.72 ± 4.52 pg/mL), 500 mg/kg (64.61 ± 2.54 pg/mL), and 1,000 mg/kg (59.17 ± 9.16 pg/mL). These results suggest a partial reduction of the acute inflammatory response. IL-6 followed a similar trend, with increased levels in the MCAO group (76.33 ± 1.17 pg/mL) compared to normal rats (42.56 ± 4.20 pg/mL). The NevG treatment experienced a notable reduction in the 250 mg/kg (49.65 ± 3.30 pg/mL), 500 mg/kg (48.11 ± 9.02 pg/mL), and 1,000 mg/kg (46.51 ± 7.05 pg/mL) groups, reflecting decreased glial activation and inflammatory load. The final pathway, NF-κβ, which is the downstream transcription factor responsible for cytokine production, was significantly increased in the MCAO group (25.06 ± 2.49 pg/mL) compared to that in normal rats (16.29 ± 1.49 pg/mL). NevG effectively inhibited NF-κB activation at all doses: 250 mg/kg (18.76 ± 1.60 pg/mL), 500 mg/kg (17.97 ± 0.63 pg/mL), and 1,000 mg/kg (15.70 ± 0.16 pg/mL). The effect of NevG was better at the highest dosage, which reduced systemic inflammation across all major inflammatory cytokine markers.

## 4 Discussion

Stroke is considered the second leading cause of death and causes disability worldwide. Ischemic stroke accounts for the highest percentage and is the most common form. Ischemic stroke is a major neurological disorder known to be the third leading cause of death in Malaysia (Abd Wahab et al., 2024). Beyond mortality, ischemic stroke results in profound physical, cognitive, and psychological impairments that significantly affect quality of life, including deficits in memory, learning, and motor function (Morozova et al., 2023). This issue is particularly relevant to Malaysia, where the proportion of the aging population is expected to increase significantly in 2030 (Sulaiman, 2024), thereby heightening the national burden of stroke-related disabilities.

There has been a growing scientific interest in medicinal mushrooms due to their wide range of bioactive compounds that possess anti-inflammatory and therapeutic effects. Research has shown that *L. rhinocerotis*, *H. erinaceus*, and *G. lucidum* can influence neuroinflammation, aid in neuronal regeneration, and enhance behavioral outcome in experimental settings. Nonetheless, most studies have focused on these mushrooms individually, with few investigations examining the potential combined therapeutic effects formulated into a single therapeutic formulation called NevG. Therefore, this study aimed to investigate whether the combination of mushrooms in NevG can reduce infarction size, improve cognitive and sensorimotor functions, and modulate inflammatory cytokines in an MCAO rat model. Addressing the outcomes provides valuable insight into the therapeutic potential of mushroom-based formulations for enhancing post-stroke recovery, especially within the aging population in Malaysia. Medicinal mushrooms have recently gained increased attention due to the diverse bioactive compounds, as *L. rhinocerotis* contains β-D-glucose, polysaccharides, flavonoids, and isoflavones that are known for anti-inflammatory activity (Genasan and Nik Ramli, 2022). There is a growing interest in the potential of *H. erinaceus* in neuroprotective action, which stimulates nerve growth factor (NGF) and enhances cognitive function through its polysaccharides, hericenones, and erinacines (Kittimongkolsuk et al., 2021). The biologically active compounds of *G. lucidum* provide neuroprotection compounds, including lectins, amino acids, lignin, mycin, and vitamins (Swallah et al., 2023). In rat models of ischemic stroke, flavonoids from the extractions of mushrooms have been shown to suppress pro-inflammatory cytokine production and reduce cerebral infarct size (Yang et al., 2024). However, most evidence remains limited to immune cell studies and selected animal models, with fewer investigations focusing on comprehensive neuroprotection mechanisms (Rowaiye et al., 2022). The beneficial effects of these mushroom extracts on ischemic stroke could play an important role in the neuroprotective efficacy in a rat MCAO model (Kong et al., 2020). Despite the benefits, most research has focused on individual species. There is still a scarcity of data on the effects of combined mushroom formulations like NevG on cognitive function, sensorimotor recovery, histological changes, and inflammatory cytokines in ischemic stroke models. Therefore, this study aimed to evaluate the potential therapeutic effects of combination mushrooms, NevG, in an MCAO rat model by examining the infarction size of ischemic brain, cognitive and sensorimotor outcomes, pyramidal cells count survival, ultrastructural integrity, and serum inflammatory cytokines.

In the present study, NevG demonstrated significant neuroprotective effects against post-ischemic injury, with the highest therapeutic effects observed at 1,000/kg over 28 days of oral treatment. This study demonstrated that NevG exerts significant neuroprotective effects against MCAO-induced ischemic stroke through multiple mechanisms involving reduced tissue damage, preserved neuronal structure, lower inflammatory responses, and improvements in both cognitive and motor outcomes.

NevG demonstrated clear dose-dependent therapeutic effects on structural, functional, and inflammatory outcomes following MCAO. Recent studies have validated the application of TTC staining to verify the degree of cerebral ischemia and the outcomes of reperfusion in the modified MCAO model (Ma et al., 2024). TTC staining confirmed that NevG reduced infarct volume in a dose-dependent manner, with the strongest effect at 1,000 mg/kg, which aligns with the tissue-preserving properties of natural bioactive compounds (Li et al., 2022). Although the staining was performed on day 29 for the treatment groups, the consistent protocol allowed for valid relative comparisons despite reduced enzyme activity in delayed staining (Kuts et al., 2019). The decrease in infarct size was linked to improved behavioral performance, supporting the idea that larger infarcts impede sensorimotor network recovery (Nur et al., 2025). Furthermore, the reduction in infarct size was linked to better neurological outcomes, implying that smaller infarct volumes are associated with less neuronal damage (Siran and Nik Ramli, 2015). This is consistent with earlier findings that MCAO-related cell death primarily impacts the striatum and cortex (Wang S.-D. et al., 2021). Histological and TEM analyses further supported these findings, where NevG-treated animals showed greater neuronal preservation, intact mitochondrial morphology, and reduced axonal damage compared to untreated MCAO rats (Ding et al., 2023; Cheng et al., 2024). NevG preserved hippocampal neurons and maintained mitochondrial and axonal integrity in a dose-dependent manner, consistent with previous findings on natural neuroprotectants (Lee et al., 2023; Xie et al., 2020). The concurrent reduction in pro-inflammatory cytokines further supports its mechanism of action, similar to the anti-inflammatory effect observed with Bu Yang Huan Wu decoction (Chen et al., 2020). Overall, these findings suggest that higher doses of NevG, particularly 500–1,000 mg/kg, provide significant long-term protection against ischemic injury through structural preservation, inflammation reduction, and functional recovery.

Neurological deficits were observed by the modified neurological severity score (mNSS) of the right limb, which is, as expected, paralyzed post-ischemic stroke, as it is contralateral to the lesion site on the left MCAO. The mNSS changes demonstrated functional improvements in deficits caused by MCAO, particularly in the limb opposite the lesion, after 28 days of treatment. The 1,000 mg/kg dosage yielded the most substantial recovery, underscoring its superior ability to preserve brain tissue and support functional recovery. These findings are consistent with previous research showing that natural anti-inflammatory compounds have therapeutic effects that effectively enhance neurological performance following an ischemic stroke. These observations were related to a previous study, which indicates that the highest dosage of wood ear mushroom significantly improved neurological deficits and suggested therapeutic effects against ischemic stroke with metabolic syndrome (Nakyam et al., 2022). Similar to existing literature, smaller infarct volume strongly correlates with reduced neurological deficits (Siran and Nik Ramli, 2015; Samuels et al., 2023). The flavonoids, polysaccharides, and terpenoid compounds found in the mushroom combinations likely contributed to reduced inflammatory signaling.

Ischemic stroke induces marked sensorimotor and cognitive deficits in rats subjected to MCAO. This study confirmed early impairments in sensorimotor function, as well as long-term deficits in both spatial and non-spatial memory following stroke (Panta et al., 2020). Previous reports indicate that the hippocampus plays a vital role in learning and episodic memory formation, and its vulnerability to ischemic injury may underlie the memory impairments frequently observed in stroke survivors, indicating a strong association between hippocampal dysfunction and post-stroke cognitive deficits. As the CA1 region in the hippocampus is particularly susceptible to ischemic damage, the observed deficits in both the MWM and T-maze reflect impaired spatial and working memory (Li et al., 2023). MCAO rats exhibited prolonged escape latency from the hidden target and increased the swimming distance, indicating impaired learning and memory similar to the previous findings, in which the MCAO model of ischemic injury can produce a deficit in hippocampal-dependent cognitive behavior (Torres-López et al., 2023). The T-maze findings revealed notable cognitive deficits in the MCAO group, as shown by lower correct choice than the normal group, highlighting memory impairments associated with the hippocampus. NevG treatment improved cognitive performance in a dose-dependent manner, with the 1,000 mg/kg group showing the greatest recovery by day 28. These improvements align with evidence that *H. erinaceus*, one component of NevG, enhances NGF expression and supports hippocampal regeneration after stroke (Cruz-Martínez et al., 2024).

Recovery of motor performance in the OFT, rotarod, and pole test further underscores the therapeutic advantages of the combination of mushrooms. In this research, MCAO rats exhibited marked impairments in locomotion, coordination, and balance, consistent with prior findings that ischemic injury disrupts motor pathways and cortical organization (Hayley et al., 2023; Singh et al., 2023). NevG treatment alleviated these motor impairments in a dose-dependent manner, with the highest dosage, 1,000 mg/kg, experiencing the most significant recovery across all assessments. Increased travel distance in the OFT implies improvements in exploratory and motor activity consistent with findings that higher therapeutic doses are more effective in mitigating ischemic motor suppression to counteract the severity of ischemic damage and support recovery after a stroke (Camargos et al., 2020). Likewise, the significant rotarod improvements suggested an improvement in coordination, endurance, and meaningful recovery of motor function. Although treated rats did not fully reach normal performance levels, the improvements suggest that NevG promotes motor recovery, likely through its neuroprotective and anti-inflammatory actions, as previously reported (Leif et al., 2021). Furthermore, the time to turn and time to descend in the pole test signify increased sensorimotor integration and descending ability, supporting NevG’s beneficial effects on sensorimotor integration (Bieber et al., 2019). Collectively, these findings demonstrate that NevG aids in restoring motor coordination following ischemic injury.

Histological and ultrastructural evaluations have revealed that the hippocampus, particularly the CA1 region, is highly susceptible to ischemic damage, showing the greatest neuronal loss, corroborating previous research on its metabolic vulnerability (Lana et al., 2022; Chauhan et al., 2021). Cresyl violet staining demonstrated significant degeneration of neurons in the CA1, CA2, CA3, and DG subregions of MCAO rats, whereas treatment with NevG preserved the neuronal viability of pyramidal cells in a therapeutic, dose-dependent manner. TEM further validated severe ischemic damage in the MCAO group, which was marked by nuclear deformation, swollen mitochondria, and disruption of axons and myelin sheaths, indicating ischemic cellular stress (Zhang et al., 2021; An et al., 2021). In this study, 90-min ligation of MCAO led to extensive loss of pyramidal neurons in the hippocampus and compromised collateral circulation in the hippocampal territory due to anatomical variations in the Circle of Willis (Lee et al., 2020). Importantly, the 1,000 mg/kg dose of NevG reduced these structural abnormalities by maintaining organelle integrity and axonal myelination. Altogether, these results align with the objectives of the study by showing that the combination of medicinal mushrooms known as NevG promotes neuronal survival and structural preservation in the hippocampus after ischemic injury.

After ischemic injury, the interruption of oxygen and glucose supply disrupts the energy of neuronal processes, making the hippocampus highly vulnerable to damage (Kafkas et al., 2024). In this study, the MCAO group marked an alteration in the nuclei, numbers of mitochondria, myelin sheath thickness, and numbers of myelinated axons, whereas NevG treatment facilitated structural restoration across all compartments. The restoration of hippocampal neurons and subcellular structures implies that NevG exerts strong therapeutic effects through mechanisms similar to those reported for natural bioactive compounds found in the combination of mushrooms. Similar research, consistent with the known actions of mushrooms, has derived bioactive compounds where polysaccharides, terpenoids, and phenolic compounds, especially from *H. erinaceus*, have been shown to enhance neuronal survival, stimulating NGF expression and minimizing inflammatory damage (Lewczuk et al., 2022; Contato and Conte-Junior, 2025). The observed improvements in mitochondrial morphology and myelin preservation align with a previous study that reported that natural triterpenes stabilize mitochondrial function and support remyelination following MCAO-induced injury (Lee et al., 2023; Feng et al., 2023). Furthermore, disrupted myelinated axons, including thinning and fragmentation of sheaths, led to impulse transmission and exacerbation post-ischemia, as highlighted by the Vagionitis and Káradóttir (2024) study on implications for myelin regeneration in recovery from ischemic stroke. By addressing mitochondrial dysfunction and myelin degeneration, the NevG treatment appears to protect the crucial subcellular structures essential for hippocampal function. These structural improvements are associated with the enhancement of cognitive and motor function, emphasizing the link between preserved hippocampal integrity and functional recovery after stroke.

A previous study has reported that ischemic stroke triggers a rapid inflammatory cascade characterized by elevated pro-inflammatory cytokines, including TNF-α, IL-1β, IL-6, and NF-κβ, which collectively exacerbate neuronal apoptosis, necrosis, and brain edema (Wang N. et al., 2021). In addition, these cytokines promote the propagation of activated macrophages, leukocytes, and macrophages in the blood cells, amplifying a neuronal injury. Serum was selected for analysis because inflammatory markers appear in circulation within 30 min after occlusion, making it a practical and clinically relevant measure (Mkhatvari, 2025). In this study, treatments with NevG remarkably reduced TNF-α, IL-1β, IL-6, and NF-κB in a dose-dependent manner, reflecting the improvements observed in neuronal preservation and behavioral outcomes. Suppression of NF-κB breaks the self-propagating inflammatory genes that exacerbate ischemia damage because it regulates multiple downstream inflammatory genes. The reduction of NF-κB and the central transcription regulating inflammatory gene expression suggest that NevG may influence NF-κB-mediated pathways, similar to other natural bioactive substances known to inhibit NF-κB activation in MCAO models (Liao et al., 2020; Guan et al., 2021). The downregulation of TNF-α, IL-1β, and IL-6 is a parallel finding in early investigations in which natural compounds, such as ginsenosides, mushroom polysaccharides, and herbal formulations, reduced these cytokines and improved ischemic outcomes. The ginsenosides have been shown to improve neurological outcomes and reduce infarct size via NF-κB suppression (Li et al., 2022), aligning with the serum concentration level of pro-inflammatory cytokines, reflecting an overall anti-inflammatory effect (Cheng et al., 2019). It is important to understand that reducing cytokine levels does not convert these molecules into anti-inflammatory agents. Their reduction modifies the immune environment after stroke, making it less inflammatory and more supportive of neuroprotection. This decrease in inflammation helps preserve neuronal integrity, limits secondary cell death, and promotes behavioral recovery in patients (Mkhatvari, 2025). Overall, the cytokine data corroborate the behavioral, histological, and ultrastructural findings, indicating suppression of pro-inflammatory cytokines contributes to reduced infarct size, improved neurological function, and increased neuronal survival following ischemic injury.

Despite the promising findings of this study, several limitations should be acknowledged to guide further research. In terms of animals, the reliance on a single sex and a relatively small group of animals limits its generalizability. Further research should include both sexes and a broader age range to capture biological diversity. A significant gap persists due to the absence of pharmacokinetic and bioavailability data of NevG, which is needed to understand its absorption and *in vivo* processing. Most importantly, the single test for each mushroom should be done on ischemic stroke in order to investigate the synergistic effects of the three mushrooms with the higher dosage. Long-term studies are necessary to evaluate its sustained effectiveness and safety in the context of chronic stroke recovery. Finally, although behavioral and histological outcomes were examined, essential neurotrophic biomarkers such as NGF, BDNF, and one specific marker in ischemic stroke were not evaluated, and their inclusion in future research would enhance mechanistic understanding.

## 5 Conclusion

This study demonstrates that NevG, a formulation of medicinal mushrooms including *L. rhinocerus, H. erinaceus*, and *G. lucidum,* provides significant neuroprotective benefits in a rat model of ischemic stroke induced by MCAO. Through a range of analyses, including the confirmation study of the brain infarction size, behavioral study, histology of pyramidal cell neurons, ultrastructural of TEM, and cytokine assessments, NevG showed therapeutic effectiveness without mortality at doses ranging from 250 mg/kg to 100 mg/kg. The highest dose of 1,000 mg/kg offered the most significant benefits, such as reduced infarct sizes, preserved neuronal structures, and improved cognitive and motor abilities, supported by a significant reduction in pro-inflammatory cytokines (TNF-α, IL-1β, IL-6, and NF-κB). These findings suggest that NevG acts through anti-inflammatory and neurotrophic pathways, likely influenced by its bioactive components that are rich in diversity of benefits, such as polysaccharides, triterpenoids, and erinacines. To conclude, the improvements in the objective parameters assessed underscore the potential of this mushroom formulation as a safe and effective natural supplement for post-stroke recovery in rats. However, the absence of comparisons between individual mushroom extracts limits the confirmation of synergistic effects. Further studies with larger sample sizes and pharmacokinetic assessments are warranted to isolate active constituents and elucidate the molecular pathways underlying the formulation of NevG through its neuroprotective efficacy.

## Data Availability

The original contributions presented in the study are included in the article/Supplementary Material; further inquiries can be directed to the corresponding authors.
